# Moderate levels of physical fitness maintain telomere length in non-senescent T CD8+ cells of aged men

**DOI:** 10.6061/clinics/2020/e1628

**Published:** 2020-11-02

**Authors:** Marta Ferreira Bastos, Manuella de Sousa Toledo Matias, Angélica Castilho Alonso, Léia Cristina Rodrigues Silva, Adriana Ladeira de Araújo, Paulo Roberto Silva, Gil Benard, Danilo Sales Bocalini, Julien Steven Baker, Luiz Eugênio Garcez Leme

**Affiliations:** I Programa de Pos graduacao Stricto sensu em Ciencias do Envelhecimento, Departamento de Pos graduacao e Pesquisa, Universidade Sao Judas Tadeu, Sao Paulo, SP, BR; II Grupo Ortogeriatrico, Instituto de Ortopedia e Traumotologia, Escola de Medicina, Universidade de Sao Paulo (FMUSP), Sao Paulo, SP, BR; III Laboratorio de Dermatologia e Imunodeficiencias, Divisao de Dermatologia, Hospital das Clinicas (HCFMUSP), Escola de Medicina, Sao Paulo, SP, BR; IV Laboratorio experimental de Fisiologia e Bioquimica, Centro de Esporte e Educacao Fisica da Universidade Federal do Espirito Santo, Vitoria, ES, BR; V Department of Sport, Physical Education and Health, Hong Kong Baptist University, Hong Kong

**Keywords:** Immunosenescence, Aging, Exercise, Telomere, T cells, CD28, Physical fitness

## Abstract

**OBJECTIVES:**

Immunosenescence is an age-associated change characterized by a decreased immune response. Although physical activity has been described as fundamental for maintaining the quality of life, few studies have evaluated the effects of different levels of exercise on telomere length in aged populations. The present study aimed to analyze the effects of different levels of physical activity, classified by the Maximal oxygen consumption (VO_2_ max) values, on the telomere length of memory Cluster of differentiation (CD) CD4^+^(CD45RO^neg^ and CD45RO^+^), effector CD8^+^CD28^neg^, and CD8^+^CD28^+^ T cells in aged individuals.

**METHODS:**

Fifty-three healthy elderly men (aged 65-85 years) were included in this study. Their fitness level was classified according to the American College of Sports Medicine (ACSM) for VO_2_ max (mL/kg/min). Blood samples were obtained from all participants to analyze the percentage of CD3, CD4, CD8, CD28^+^, naïve, and subpopulations of memory T cells by using flow cytometry. Furthermore, using the Flow-FISH methodology, the CD4^+^CD45RO^+^, CD4^+^CD45RO^neg^, CD8^+^CD28^+^, and CD8^+^CD28^neg^T cell telomere lengths were measured.

**RESULTS:**

There was a greater proportion of effector memory T CD4^+^ cells and longer telomeres in CD8^+^CD28^+^ T cells in the moderate physical fitness group than in the other groups. There was a higher proportion of terminally differentiated memory effector T cells in the low physical fitness group.

**CONCLUSION:**

A moderate physical activity may positively influence the telomere shortening of CD28^+^CD8^+^T cells. However, additional studies are necessary to evaluate the importance of this finding with regard to immune function responses in older men.

## INTRODUCTION

Aging is a complex process characterized by a series of changes in an organism. It may be associated with an imbalance between damage and tissue repair, which is responsible for a degeneration phenotype mediated by environmental and genetic factors. Furthermore, the age-associated changes in the ability of tissues to replace the loss or damage of cells may be associated with several age-related diseases (1).

Telomeres are the regions located at the end of the eukaryote chromosomes and are responsible for protecting the genomic Deoxyribonucleic acid (DNA) from enzymatic degradation. In addition, changes in telomere lengths are considered well-established markers of the aging process, as these regions shorten with each cell division (2). Therefore, an increase in chronological age can promote telomere shortening, and this change has been associated with senescence, as well as senility (1,3).

Immunosenescence is an age-associated alteration in the immune system and is characterized by a decline of the innate and adaptive immunity, which is associated with an increase in the susceptibility to infections, autoimmune diseases, and cancer (4). The main age-associated changes described are related to T cell activity. T cells are produced in the bone marrow, maturated in the thymus, entering the circulation, and being transferred to peripheral lymphoid tissues (5,6). The involution of the thymus is a natural event observed with aging and is associated with a progressive reduction in the reposition of naïve CD4^+^ and CD8^+^T cells, which provide a lower capacity for elderly individuals to respond to novel infectious agents (7,8). Memory cells are formed after antigen recognition and present the ability to survive in a quiescent state for long periods. They can also rapidly respond to the exposure to new antigens (9). It has been proposed that the differentiation from naïve to memory T cells is a complex process, in which distinct memory T cell subpopulations are formed. These remain in circulation or migrate to peripheral tissues and have the potential to become senescent cells (10,11). Three distinct subpopulations of memory T cells have been described: central memory T cells (TCM cells), effector memory T cells (TEM cells), and terminally differentiated memory effector T cells, which are T cells that re-express Cluster of differentiation (CD) CD45RA molecule (3).

Physical activity has been described as fundamental for maintaining the quality of life and decreasing the incidence or severity of diseases in aged subjects (12). Although some studies have shown that a chronic physical activity is associated with the preservation of telomere lengths in leukocytes (13-15,3), only a few studies have been conducted to investigate the effects of different levels of physical activity on T cell expression markers of immunosenescence. The impact of physical exercise on the telomere lengths of T cells needs to be further investigated, since the telomere lengths of T cells could preserve immune function in older adults.

Several markers are used to characterize memory T cells, among which CD45RO has been more frequently associated with the aging process; the proportion of memory T cells has been widely described, compared to that of naïve T cells (16,17,3). Another key marker to evaluate the aging process of T cells is CD28. This is an essential co-stimulatory molecule present in several types of T cells and is responsible for the activation and survival of these cells (18,19). Therefore, the populations of CD28^neg^ T cells are considered to be senescent cells; among them, CD8^+^ T cells were the first population to exhibit a shortening of telomeres and to show a decreased immune response in aged individuals. This response is associated with cancer, viral infections, autoimmunity, and almost every chronic inflammatory disease (20-22).

The aim of the present study was to analyze the proportion of memory T cells and to evaluate the telomere lengths of memory T CD4^+^ (CD45RO^+^ and CD45RO^neg^) and senescent CD28^+^ CD8^+^ T cells in elderly individuals with different levels of physical activity, classified according to the Maximal oxygen consumption (VO_2_ max values).

## MATERIAL AND METHODS

### Participants

The study protocol was explained to each subject, and their signed informed consent was obtained. The Ethics Committee at the Faculty of Medicine, University of Sa?o Paulo (registration number 0135/11) approved the protocol of this study, according to the principles of the Declaration of Helsinki. Fifty-three healthy elderly men (65 to 85 years) were recruited mainly from running associations, sports clubs, and community-based exercise programs for elderly outpatient services of São Paulo city (São Paulo, Brazil). Most of the participants were runners, including short distance and marathon runners, bodybuilders, soccer players, dancers, walkers, cyclists, volleyball players, basketball players, and engaged in the general physical activities provided to the elderly in public health programs. The subjects were classified according to the level of physical fitness, as outlined by the American College of Sports Medicine (23) regarding VO_2_ max values (mL/kg/min). Based on this classification, the participants were divided into three groups: low group (n=23), with subjects with low or very low physical fitness; moderate group (n=7), that is, the subjects who participated in physical activity two or three times/week, and high group (n=23), composed of subjects exhibiting a good or excellent physical fitness level.

The exclusion criteria were co-morbidities that could interfere with immune system function (e.g., Acquired ImmunoDeficiency Syndrome (AIDS), cancer, rheumatoid arthritis, and uncontrolled diabetes mellitus), use of immunosuppressive drugs (e.g., corticosteroids), smoking, and/or alcohol abuse.

### Procedures

All participants were submitted to clinical evaluation by a physician. Baseline collected data included body mass (kg) and height (m), which were used to calculate the body mass index (BMI, kg/m^2^). Blood samples were collected 48 h after the last regular training session and, at least, one week after participation in any competition. Blood samples were used to evaluate naïve (C-C chemokine receptor type 7 (CCR7^+^)CD45RA^+^), and the subpopulations of memory T cells: central memory (TCM, CCR7^+^CD45RA^neg^), effector memory (TEM, CCR7^neg^CD45RA^neg^), and terminally differentiated effector memory T cells (TEMRA, CCR7^neg^CD45RA^+^), as well as to analyze the telomere lengths of CD4^+^CD45RO^+^, CD4^+^CD45RO^neg^, CD8^+^CD28^+^, and CD8^+^CD28^neg^ T cells.

### Measurement of VO_2_ max consumption

The fitness status was assessed using a treadmill (hp/Pulsar, Cosmos, Germany), and by collecting the expired gas, to calculate VO_2_ max volumes. Briefly, during cardiopulmonary exercise testing, individuals were monitored using a computerized electrocardiograph (HeartWare Instruments, Ergo 13, Brazil), and the heart rate was measured using Electrocardiogram (ECG) recording (lead I (DI), lead (DII), lead III (DIII), augmented Voltage foot (AVf), augmented Voltage left arm (AVl), augmented Voltage right arm (AVr) and fourth intercostal space, to the right sternum (V1), fourth intercostal space, to the left sternum (V2), placed diagonally between Ve and V4 (V3), between rib 5 and 6 in midclavicular line (V4), placed on the same level as V4, but in the anterior axillary line (V5), placed on the same level as V4 and V5, but in the midaxillary line (V6)) at rest, during exercise, and during the recuperation phase at the end of each minute. Oxygen consumption (VO_2_), carbon dioxide output (VCO_2_), and minute ventilation (VE) were measured and analyzed using a calibrated metabolic analyzer (CPX/D, MedGraphics, USA) (24). A modified version of the Heck stress test protocol was used (32). The VO_2_ max values calculated were used to categorize the subjects into three groups according to the physical status previously described by ACSM (25). All data were collected at the same time of the day to avoid diurnal contamination.

### Subpopulations of T cells

Blood samples were collected and labeled using a mix of monoclonal antibodies for 20 min at room temperature: anti-CD3 V500, anti-CD4 V450, anti-CD8 Antigen-presenting cell cyanine dye (APC-Cy7), anti-CD45RA fluorescein isothiocyanate (FITC), and anti-CCR7 Phycoerythrincyanine dye (PE-Cy7) (BD Biosciences, San Diego, California (CA), USA). The erythrocytes were depleted with OptiLyse C (Beckman Coulter, CA, USA), washed, suspended in BD FACS Flow solution (BD Biosciences, CA, USA), and analyzed using Fluorescenceactivated cell sorting (FACS) Fortessa, FACS Diva software (BD Biosciences, CA, USA), and FlowJo software (version 7.4, Tree Star, San Carlos, CA). Fluorescence voltages were determined using matched unstained cells. Compensation was performed using single-stained CompBeads (BD Biosciences, CA, USA). Data from samples were acquired until at least 200,000 events were collected in a live lymphocyte gate.

### Telomere length measurement using Flow Fish

Peripheral blood mononuclear cells (PBMCs) were isolated from heparinized blood using density gradient centrifugation and Ficoll-Paque Plus (GE Healthcare Life Sciences, Little Chalfont, UK). CD4^+^ and CD8^+^ T cell subsets were negatively selected using magnetic microbeads and Magnetic cell separation strategies (MACS) Cell Separation Reagents (Miltenyi Biotec, Germany), according to the manufacturer’s instructions. CD45RO and CD28 were purified using microbeads (Miltenyi Biotec, Germany) to obtain CD45RO^+^ or CD45RO^neg^CD4^+^ T cells and CD28^+^ or CD28^neg^CD8^+^ T cells. The telomeres of these populations were analyzed using the Telomere peptide nucleic acid (PNA) kit/FITC (Dako, CO, UK), according to the manufacturer’ s recommendations, as previously described (Derradji et al. 2005). Briefly, the DNA from these cells or control cells (cell line 1301) was denatured for 10 min at 82°C in hybridization solution with or without a FITC-conjugated PNA telomere probe, and then hybridized overnight at 23°C. Subsequently, the samples were washed twice for 10 min using a Wash Solution at 40°C and resuspended in DNA staining solution to identify cells in the G0/1 phase. Samples were acquired on a FACS Fortessa using FACS Diva software (BD Biosciences, CA, USA) and analyzed using FlowJo software (version 7.4, Tree Star, San Carlo, CA). The relative telomere length (RTL) was calculated as the ratio of the telomere signals (FITC fluorescence) of the sample and the control 1301 cell line, with the correction for the DNA index of G0/1 cells, and converted to kilobase pairs using the telomere length of 1301 cell line (23,480 bp) (26).

### Statistical analyses

Statistical analyses were performed using GraphPad Prism 8.0 (GraphPad Software Inc., San Diego, CA, USA). Prior to statistical analysis, data were first examined for normality using the Shapiro Wilk test. Further analysis was performed using the Kruskal-Wallis test in conjunction with Dunn's *post-hoc* test. The results were expressed as median and/or minimum (min) and maximum (max) with the significance level established at 5%.

## RESULTS

The participants were classified into three groups, according to the physical fitness level: low, moderate, and high. No significant differences were detected in age and anthropometric parameters (body mass, height, and body mass index) among the groups (*p*>0.05, Table 1).

As expected, there was a significant difference in the VO_2_ max levels among all groups (*p*<0.001). Oxygen consumption in the low physical fitness level group was lower (24.6 mL/kg/min) than in the moderate (31 mL/kg/min) and high groups (35.5 mL/kg/min).

The percentage of subpopulations of CD4^+^ and CD8^+^ T cells was determined using flow cytometry. Initially, no differences were reported in the values of CD4^+^ (*p*=0.29), CD8^+^ (*p*=0.34), and the CD4/CD8 ratio (*p*=0.36) among the groups. The median values of CD4^+^, CD8^+^, and CD4/CD8 ratios were, respectively: 57.1, 33, and 1.6 for the low; 46.9, 34.7, and 1.3 for the moderate; and 60.5, 30.2, and 2.0 for the high physical fitness groups. These results demonstrated that different levels of chronic physical exercise did not moderate the proportion of CD4 and CD8 lymphocytes, which represent very important cells in adaptive immune responses. In addition, no differences were observed in the proportion of the CD28^+^ marker for CD4 and CD8 T cells, which supports the absence of differences in the proportion of senescent cells among the participants of the present study.

**Figure 1:**
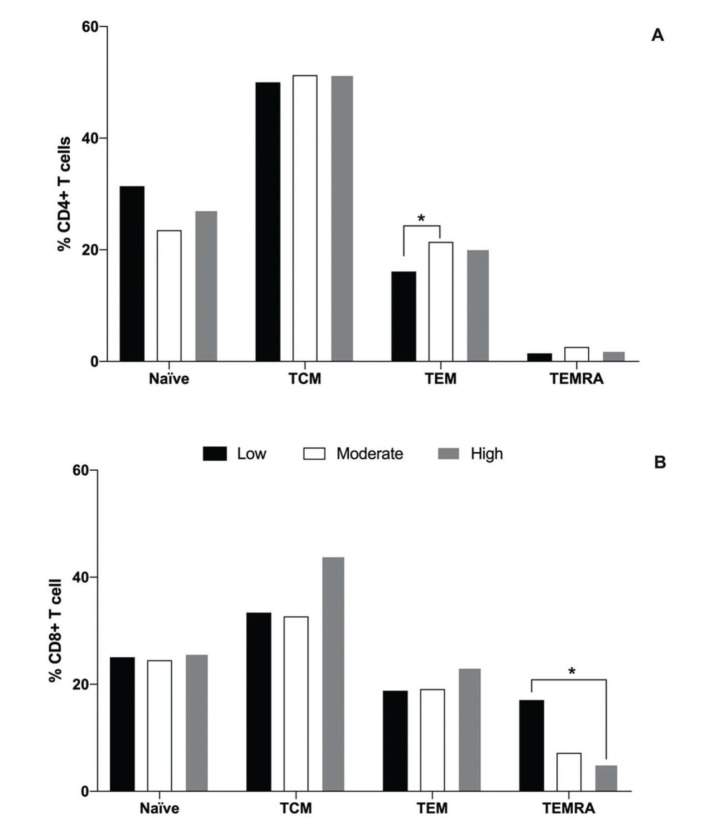
Median percentages of naïve (CCR7^+^CD45RA^+^), central memory (TCM, CCR7^+^CD45RA^neg^), effector memory (TEM, negative to C-C chemokine receptor type 7 (CCR7^neg^) negative to Cluster of differentiation type 45RA (CD45RA^neg^), and effector memory RA s(CCR7^neg^CD45RA^+^) cells of the CD4^+^ (A) and CD8^+^ (B) T cells for the low (black bar), moderate (white bar), and high (gray bar) physical fitness groups. *represents statistical difference among the groups, as evaluated using Kruskal-Wallis and Dunn’s *post-hoc* tests (*p*<0.05).

With regard to the naïve and subpopulations of memory T cells, it was observed that the moderate group had a greater percentage of TEM CD4 cells (21.4%) than the low physical fitness level group (16.1%, *p*=0,04), while the high group presented an intermediate percentage (20.2%), as illustrated in Figure 1A. No differences were observed in the percentage of naïve, TCM, and TEMRA CD4^+^ cells among all groups. With regard to CD8^+^ T cells, it was observed that the low physical fitness group exhibited a greater percentage of TEMRA cells (18.8%) than the high physical fitness level group (5.1%, *p*<0.001), and the percentages of these cells in low and high physical fitness level groups were similar to that in the moderate group (7.2%). No differences were detected in the percentage of naïve, TCM, and TEM CD8^+^ cells among all groups (Fig. 1B).

**Figure 2:**
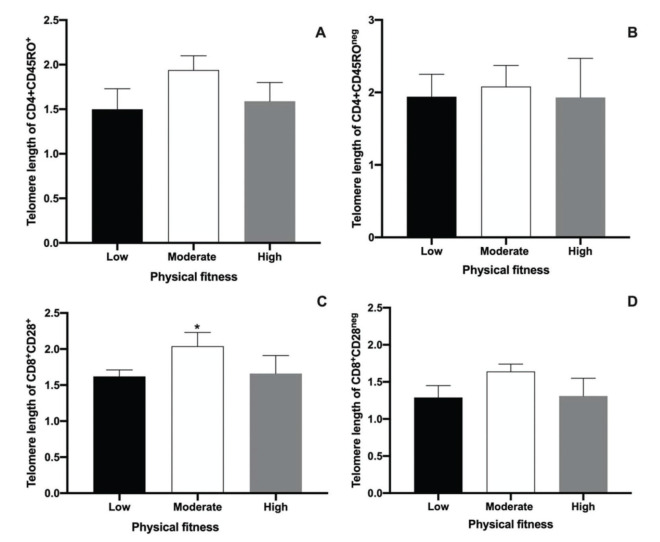
Median and interquartile ranges of telomere length measurements performed using Flow-Fish in CD4^+^CD45RO^+^ (A), CD4^+^CD45RO^neg^(B), CD8^+^CD28^+^(C), and CD8^+^CD28^neg^ cells (D). Results are presented as the median and interquartile ranges. * represents significant differences analyzed using Kruskal-Wallis and Dunn’s *post-hoc* tests (*p*<0.01).

The main objective of the present study was to perform an analysis of the telomere lengths of different T cell populations (CD4^+^CD45RO^+^ and CD4^+^CD45RO^neg^, CD8^+^CD28^+^, and CD8^+^CD28^neg^) using Flow-Fish. The results are shown in Figure 2. No differences were observed among low, moderate, and high physical fitness groups with regard to the telomere length in CD4^+^CD45RO^+^ and CD4^+^CD45RO^neg^ T cells. This result demonstrates the absence of the effects of physical fitness on the telomere length of memory CD4^+^T cells in aged men. The median values ??of the telomere lengths observed in the low, moderate, and high groups for the CD4^+^CD45RO^+^ T cell population were 1.5, 1.94, and 1.59 kbp, respectively (Fig. 2A). In CD4^+^CD45RO^neg^ cell populations, the median telomere length was 1.94, 2.08, and 1.93 kbp for the low, moderate, and high physical fitness groups, respectively (Fig. 2B).

The present study also evaluated telomere length in senescent CD8^+^T cells in aged men with different levels of physical fitness using the CD28 marker. Although no differences in the telomere length of CD8^+^CD28^neg^ T cells were detected among the groups (Fig. 2D), a significant difference in the telomere lengths of CD8^+^CD28^+^ T cells was observed. A greater telomere length in the moderate physical fitness group was also noted, compared to that in the other groups (Fig. 2C, *p*<0.0035). The median telomere length values of the CD8^+^CD28^+^ T cell population were 1.62, 2.04, and 1.66 kbp in the low, moderate, and high physical fitness groups, respectively (Fig. 2C). In the CD8^+^CD28^neg^ T cell population, the telomere lengths were 1.29, 1.64, and 1.31 kbp for the low, moderate, and high physical fitness groups, respectively (Fig. 2D).

## DISCUSSION

Immunosenescence is a natural process characterized by the loss of naïve T cells and the accumulation of memory T cells, resulting from contact with pathogens over the years (27). In addition, the loss of CD28 expression, telomere shortening, and the re-expression of CD45RA are phenotypic changes in T cells that lead to harmful functional alterations, such as decreased proliferative responses, insufficient IL-2 synthesis, and apoptosis resistance (28-32). The important impact of immunosenescence on the health status of aged subjects has been previously recognized, and the discovery of alternative treatments or habits that favor the quality of immune responses are of fundamental importance (33). Physical exercise has been described as an alternative, inexpensive, and safe intervention to reduce the negative impacts of immunosenescence (34-36).

In the present study, we evaluated whether different levels of physical fitness could alter the proportion of CD4, CD8, CD28^+^, CD28^neg^, naïve, and memory T cells in aged men. No differences were reported with regard to the proportion of CD4 and CD8 T cell subpopulations, demonstrating that different levels of physical activity do not affect the CD4/CD8 ratio among the groups investigated. A CD4/CD8 ratio up to 1.0, as observed for all participants in the present study, has been associated with a lower mortality in older adults. This result suggests that physical practice may control at least one of the parameters of immunological risk (17,37,3). In addition, the proportion of CD28^+^ and CD28^neg^ cells did not change in older men with different levels of physical fitness, and since CD28 is considered an important aging biomarker (38), these data suggests that physical activity, independent of the level, could maintain the proportions of circulating non-senescent T cells in aged men. Unfortunately, only a few studies have evaluated the relationship between physically active lifestyles and immunosenescence in older adults. In a systematic review, Dihn et al. (36) reported that studies on exercise and immunosenescence demonstrated that acute physical exercise could increase the levels of senescent cells in younger or middle-aged adults. It is important to highlight that senescent T cells are more resistant to apoptosis, incapable of dividing, and the major source of chronic inflammatory profiles (39,40). Thus, further studies are necessary to evaluate the impact of physical exercise on senescent T cells in older adults and its contribution to the maintenance of a favorable health status.

Moreover, in the present study, we observed that different levels of physical fitness did not alter the percentage of naïve CD4 and CD8 T cells, although a lower percentage of naïve cells was detected, compared to memory T cells. This finding is in agreement with the literature, and this decline in peripheral naïve T lymphocytes is associated with an impaired function of the immune system that is used to combat novel pathogens (41-43). Regarding the memory T cell subpopulation, the moderate physical fitness group exhibited a higher proportion of TEM CD4^+^ cells, when compared with the low fitness group; these cells represent a subpopulation that showed an immediate effector function, but only a limited proliferative potential. In addition, the low physical fitness group showed greater proportions of TEMRA CD8^+^ cells compared to the high fitness group, which supports the importance of physical exercise in modulating the cell profile associated with an increased immunological risk in older adults (34,35). To the best of our knowledge, thus far, only two studies have focused on the association of different physical fitness levels and the proportion of memory T cell subpopulations, and these studies partially corroborate the results of the present study. Silva et al. (3) tested the hypothesis that a moderate or intense physical exercise intervention in an elderly group could attenuate the effects of aging on telomere length and the composition of T cell subsets, compared to a non-active elderly group. It was demonstrated that a greater proportion of TEM CD8^+^ in the intense exercise group and a lower proportion of TEMRA CD4^+^ and CD8^+^ cells were observed in the training groups, compared to that in the non-trained group. This suggests that moderate and intense exercise lifestyles attenuate some of the effects of aging with regard to the composition and survival of memory T cell subpopulations. Minuzzi et al. (44) evaluated the effects of lifelong training on the senescence and mobilization of T lymphocytes in response to acute exercise in master athletes (53.7 years and VO_2_ max=40.3 mL/kg/min) characterized by regular physical training over a 20-year period. Although there were differences in the participant profiles, the above-mentioned study also demonstrated a lower percentage of senescent TEMRA CD4^+^ and CD8^+^ T cells in the master athletes. TEMRA cells are considered the most antigen-experienced cells, characterized by a reduced proliferative capacity and a high sensitivity to apoptosis, and are linked to the loss of immunological efficiency of an aged immune system (43), which emphasizes the important benefits of physical exercise on the immune system function in older adults.

In the present study, we also evaluated if different levels of physical activity could affect telomere shortening in aged men. There was absence of the differences in the telomere length for classical memory marker (CD45RO) in CD4+T cells among individuals with low, moderate and high levels of physical fitness. Although several previous studies have evaluated the effects of physical activity on telomere length, the results remain controversial, both for analyses performed on muscle cells and leukocytes. Collins et al. (45) demonstrated a shortening of the telomeres of muscle cells in athletes and suggested a possible mechanism related to the impact of stress, promoted by physical exercise on the musculoskeletal system. On the other hand, Rae et al. (46) demonstrated that athletes and sedentary individuals presented similar telomere lengths in the cells of the musculoskeletal system. Magi et al. (47) demonstrated that skeletal muscles from healthy adults display an age-dependent telomere attrition rate, which is partially influenced by exercise. In turn, studies that evaluate telomere lengths in immune-related cells are also conflicting. Ludlow et al. (1) compared good, excellent, and low or very low physical training with regard to immune function in subjects aged 50 to 70 years and suggested that moderate physical fitness levels have been associated with a protective effect on telomere length in the peripheral blood mononuclear cells of aged subjects. Savela et al. (48) has associated physical activity with telomere length in middle-aged (47 years) and elderly (76 years) subjects. After a 29-year follow-up, it was observed that the frequency of short telomeres was the lowest in the moderate physical activity group, when compared with that in the high and low physical activity groups. The authors concluded that both a low and high physical activity are associated with a shortening of leukocyte telomere length. Cherkas et al. (15) evaluated a small group of twins for their physical activity levels and demonstrated that the twins that were more active presented a longer telomere length than the less active twins. Tucker (49) showed that adults who achieved high levels of physical activity tended to have longer telomeres compared to their more sedentary counterparts. Similar findings were reported by Shadyab et al. (50), who evaluated 1,476 older white and African American women in a Women's Health Initiative Objective Physical Activity and Cardiovascular Health study. However, in a cross-sectional study including 588 participants, Ding et al. (51) described a non-significant association between physical activity and telomere length in a Northern Chinese population. These divergences among studies are due to the differences in the experimental designs, immunological parameters, participant profiles, and the type, intensity, and regularity of physical activity.

The loss of CD28 markers on lymphocytes has been strongly associated with age-related adaptive immunity, especially to effector CD8^+^ T cells and is considered an important biomarker of immunosenescence (32,52). Interestingly, the present study showed a longer telomere length in CD28^+^CD8^+^ T cells of aged men that had a moderate level of physical activity, compared with subjects who had high and low levels of physical activity. Consistent with the present study, Silva et al. (3) also detected a longer telomere in CD8^+^CD28^neg^ T cells in an intensively trained group when compared to the case in non-trained groups. They hypothesized that middle-aged track-and-field athletes could have reduced telomere erosion compared with non-athletes, probably due to an augmented expression of telomere-stabilizing proteins and an increased telomerase activity (53). Again, these findings highlight the importance of promoting health behaviors as regular practices to diminish the risk of aged-related diseases. However, Mundstock et al. (54) concluded that there is insufficient evidence to associate physical activity with telomere shortening. In addition, Chilton et al. (55) concluded that leukocyte telomere length is highly variable and may be influenced by genetic characteristics, gender, and paternal age at conception. In addition, telomere lengths also undergo lifelong exposure to oxidative and inflammatory damage.

Some limitations of this study should be mentioned, such as the use of other phenotypical markers and modern methods to analyze telomere lengths. Moreover, a higher number of subjects enrolled in the moderate group, which was lower compared to the other groups, would improve the statistical analysis and limit the intensity and duration variations, which may be considered as a potential bias in the present study.

## CONCLUSION

In conclusion, moderate physical activity seems to prevent telomere shortening in CD28^+^CD8^+^T cells, which are an important cell type related to immune effectiveness in aged populations. Furthermore, additional studies are necessary to further evaluate the importance of this finding on the immune response of older men to different exercise regimes and fitness levels.

## AUTHOR CONTRIBUTIONS

Matias MS, Silva LC, Alonso AC, Bastos MF and Araujo AL were responsible for the recruitment of the participants, blood sampling and processing of the data. Matias MS and Leme LE were responsible for clinical evaluation of the participants. Alonso AC and Bocalini DS were responsible for the physical evaluation of the participants and Silva PR was responsible for the treadmill VO2 max consumption test. Benard G and Leme LE designed and coordinated the study. Benard G, Leme LE and Baker JS were responsible for manuscript review and Bastos MF was responsible for manuscript writing, editing and review.

## Figures and Tables

**Table 1:** Age and anthropometric data of participants.

Parameters	Physical fitness groups		
	Low (n=23)	Moderate (n=7)	High (n=23)
Age (years)	68 (66-75)	69 (65-70)	73 (66-74)
Body mass (kg)	74 (66-79)	71 (62-77)	66.5 (58-73)
Height (m)	1.7 (1.5-1.8)	1.7 (1.65-1.73)	1.67 (1.63-1.73)
Body mass index (kg/m^2^)	25 (23.8-26.3)	25 (23.6-25.9)	23 (21.9-24.5)
VO_2_ max (mL/kg/min)	24.6† (22.3-27.1)	31# (28.4-32.5)	35.5* (31.0-36.9)

Values are expressed as the medians (Min-Max). Different symbols represent statistically significant differences detected using Kruskal-Wallis and Dunn’s *post-hoc* tests.

† Significant difference between the moderate and low groups (*p*=0.001).

* Significant difference between the high and low groups (*p*<0.001).

# Significant difference between the high and moderate groups (*p*<0.001).
